# Supplementary material for: Iterative improvement in the automatic modular design of robot swarms
Source: PeerJ Comput Sci. 2020 Dec 7;6:e322. doi: 10.7717/peerj-cs.322 (PMC7924708; doi:10.7717/peerj-cs.322)
Supplement: Supplemental Information 3 [file peerj-cs-06-322-s003.zip › argos3/doc/api/standalone/a00402_source.html]

ARGoS: core/utility/profiler/profiler.cpp Source File


- Main Page
- Related Pages
- Namespaces
- Classes
- Files

- File List
- File Members

# core/utility/profiler/profiler.cpp

Go to the documentation of this file.

```
00001 
00007 #include "profiler.h"
00008 #include <argos3/core/utility/logging/argos_log.h>
00009 #include <argos3/core/utility/configuration/argos_configuration.h>
00010 
00011 namespace argos {
00012 
00013    /****************************************/
00014    /****************************************/
00015 
00016    static double TV2Sec(const ::timeval& t_timeval) {
00017       return
00018          static_cast<double>(t_timeval.tv_sec) +
00019          static_cast<double>(t_timeval.tv_usec) * 0.000001;
00020    }
00021 
00022    /****************************************/
00023    /****************************************/
00024 
00025    static double CPUUsage(const ::rusage& t_rusage,
00026                           double f_elapsed_time) {
00027       return 100.0 *
00028          (TV2Sec(t_rusage.ru_utime) +
00029           TV2Sec(t_rusage.ru_stime)) /
00030          f_elapsed_time;
00031    }
00032 
00033    /****************************************/
00034    /****************************************/
00035 
00036 #define DUMP_RUSAGE_FIELD_HUMAN_READABLE(MSG, FIELD) c_os << MSG ": " << t_resources.ru_ ## FIELD << std::endl;
00037    void DumpResourceUsageHumanReadable(std::ostream& c_os,
00038                                        const ::rusage& t_resources) {
00039       double fUserTime = TV2Sec(t_resources.ru_utime);
00040       c_os << "User time: " << fUserTime << std::endl;
00041       double fSysTime = TV2Sec(t_resources.ru_stime);
00042       c_os << "System time: " << fSysTime << std::endl;
00043       DUMP_RUSAGE_FIELD_HUMAN_READABLE("Maximum resident set size", maxrss);
00044       DUMP_RUSAGE_FIELD_HUMAN_READABLE("Integral shared memory size", ixrss);
00045       DUMP_RUSAGE_FIELD_HUMAN_READABLE("Integral unshared data size", idrss);
00046       DUMP_RUSAGE_FIELD_HUMAN_READABLE("Integral unshared stack size", isrss);
00047       DUMP_RUSAGE_FIELD_HUMAN_READABLE("Page reclaims", minflt);
00048       DUMP_RUSAGE_FIELD_HUMAN_READABLE("Page faults", majflt);
00049       DUMP_RUSAGE_FIELD_HUMAN_READABLE("Swaps", nswap);
00050       DUMP_RUSAGE_FIELD_HUMAN_READABLE("Block input operations", inblock);
00051       DUMP_RUSAGE_FIELD_HUMAN_READABLE("Block output operations", oublock);
00052       DUMP_RUSAGE_FIELD_HUMAN_READABLE("Messages sent", msgsnd);
00053       DUMP_RUSAGE_FIELD_HUMAN_READABLE("Messages received", msgrcv);
00054       DUMP_RUSAGE_FIELD_HUMAN_READABLE("Signals received", nsignals);
00055       DUMP_RUSAGE_FIELD_HUMAN_READABLE("Voluntary context switches", nvcsw);
00056       DUMP_RUSAGE_FIELD_HUMAN_READABLE("Involuntary context switches", nivcsw);
00057    }
00058 
00059    /****************************************/
00060    /****************************************/
00061 
00062 #define DUMP_RUSAGE_FIELD_TABLE_ROW(FIELD) c_os << " " << t_resources.ru_ ## FIELD;
00063    void DumpResourceUsageAsTableRow(std::ostream& c_os,
00064                                     const ::rusage& t_resources) {
00065       double fUserTime = TV2Sec(t_resources.ru_utime);
00066       c_os << fUserTime;
00067       double fSysTime = TV2Sec(t_resources.ru_stime);
00068       c_os << " " << fSysTime;
00069       DUMP_RUSAGE_FIELD_TABLE_ROW(maxrss);
00070       DUMP_RUSAGE_FIELD_TABLE_ROW(ixrss);
00071       DUMP_RUSAGE_FIELD_TABLE_ROW(idrss);
00072       DUMP_RUSAGE_FIELD_TABLE_ROW(isrss);
00073       DUMP_RUSAGE_FIELD_TABLE_ROW(minflt);
00074       DUMP_RUSAGE_FIELD_TABLE_ROW(majflt);
00075       DUMP_RUSAGE_FIELD_TABLE_ROW(nswap);
00076       DUMP_RUSAGE_FIELD_TABLE_ROW(inblock);
00077       DUMP_RUSAGE_FIELD_TABLE_ROW(oublock);
00078       DUMP_RUSAGE_FIELD_TABLE_ROW(msgsnd);
00079       DUMP_RUSAGE_FIELD_TABLE_ROW(msgrcv);
00080       DUMP_RUSAGE_FIELD_TABLE_ROW(nsignals);
00081       DUMP_RUSAGE_FIELD_TABLE_ROW(nvcsw);
00082       DUMP_RUSAGE_FIELD_TABLE_ROW(nivcsw);
00083    }
00084 
00085    /****************************************/
00086    /****************************************/
00087 
00088 #define DIFF_RUSAGE_FIELD(FIELD) tResult.ru_ ## FIELD = t_resource1.ru_ ## FIELD - t_resource2.ru_ ## FIELD;
00089    ::rusage operator-(const ::rusage& t_resource1,
00090                       const ::rusage& t_resource2) {
00091       ::rusage tResult;
00092       DIFF_RUSAGE_FIELD(utime.tv_sec);
00093       DIFF_RUSAGE_FIELD(utime.tv_usec);
00094       DIFF_RUSAGE_FIELD(stime.tv_sec);
00095       DIFF_RUSAGE_FIELD(stime.tv_usec);
00096       DIFF_RUSAGE_FIELD(maxrss);
00097       DIFF_RUSAGE_FIELD(ixrss);
00098       DIFF_RUSAGE_FIELD(idrss);
00099       DIFF_RUSAGE_FIELD(isrss);
00100       DIFF_RUSAGE_FIELD(minflt);
00101       DIFF_RUSAGE_FIELD(majflt);
00102       DIFF_RUSAGE_FIELD(nswap);
00103       DIFF_RUSAGE_FIELD(inblock);
00104       DIFF_RUSAGE_FIELD(oublock);
00105       DIFF_RUSAGE_FIELD(msgsnd);
00106       DIFF_RUSAGE_FIELD(msgrcv);
00107       DIFF_RUSAGE_FIELD(nsignals);
00108       DIFF_RUSAGE_FIELD(nvcsw);
00109       DIFF_RUSAGE_FIELD(nivcsw);
00110       return tResult;
00111    }
00112 
00113    /****************************************/
00114    /****************************************/
00115 
00116    CProfiler::CProfiler(const std::string& str_file_name,
00117                         bool b_trunc) {
00118       if(b_trunc) {
00119          m_cOutFile.open(str_file_name.c_str(),
00120                          std::ios::trunc | std::ios::out);
00121       }
00122       else {
00123          m_cOutFile.open(str_file_name.c_str(),
00124                          std::ios::app | std::ios::out);
00125       }
00126       LOG << "Opened file \"" << str_file_name << "\" for profiling." << std::endl;
00127       int nError = pthread_mutex_init(&m_tThreadResourceUsageMutex, NULL);
00128       if(nError) {
00129          THROW_ARGOSEXCEPTION("Error creating thread profiler mutex " << ::strerror(nError));
00130       }
00131    }
00132 
00133    /****************************************/
00134    /****************************************/
00135 
00136    CProfiler::~CProfiler() {
00137       m_cOutFile.close();
00138       pthread_mutex_destroy(&m_tThreadResourceUsageMutex);
00139    }
00140 
00141    /****************************************/
00142    /****************************************/
00143 
00144    void CProfiler::Start() {
00145       StartWallClock();
00146       StartCPUProfiler();
00147    }
00148 
00149    /****************************************/
00150    /****************************************/
00151 
00152    void CProfiler::Stop() {
00153       StopCPUProfiler();
00154       StopWallClock();
00155    }
00156 
00157    /****************************************/
00158    /****************************************/
00159 
00160    void CProfiler::Flush(bool b_human_readable) {
00161       if(b_human_readable) {
00162          FlushHumanReadable();
00163       }
00164       else {
00165          FlushAsTable();
00166       }
00167    }
00168 
00169    /****************************************/
00170    /****************************************/
00171 
00172    void CProfiler::CollectThreadResourceUsage() {
00173       pthread_mutex_lock(&m_tThreadResourceUsageMutex);
00174       ::rusage tResourceUsage;
00175 #ifdef __APPLE__
00176       /* FIXME: This is badly broken, but I don't know how to obtain
00177          the equivalent of RUSAGE_THREAD on MacOS. --tc */
00178       getrusage(RUSAGE_SELF, &tResourceUsage);
00179 #else
00180       ::getrusage(::RUSAGE_THREAD, &tResourceUsage);
00181 #endif
00182       m_vecThreadResourceUsage.push_back(tResourceUsage);
00183       pthread_mutex_unlock(&m_tThreadResourceUsageMutex);
00184    }
00185 
00186    /****************************************/
00187    /****************************************/
00188 
00189    void CProfiler::FlushHumanReadable() {
00190       m_cOutFile << "[profiled portion overall]" << std::endl << std::endl;
00191       double fStartTime = TV2Sec(m_tWallClockStart);
00192       double fEndTime = TV2Sec(m_tWallClockEnd);
00193       double fElapsedTime = fEndTime - fStartTime;
00194       m_cOutFile << "Wall clock time: " << fElapsedTime << std::endl;
00195       ::rusage tDiffResourceUsage = m_tResourceUsageEnd - m_tResourceUsageStart;
00196       m_cOutFile << "CPU usage: " << CPUUsage(tDiffResourceUsage, fElapsedTime) << "%" << std::endl;
00197       DumpResourceUsageHumanReadable(m_cOutFile, tDiffResourceUsage);
00198       m_cOutFile << std::endl << "[profiled portion start]" << std::endl << std::endl;
00199       DumpResourceUsageHumanReadable(m_cOutFile, m_tResourceUsageStart);
00200       m_cOutFile << std::endl << "[profiled portion end]" << std::endl << std::endl;
00201       DumpResourceUsageHumanReadable(m_cOutFile, m_tResourceUsageEnd);
00202       m_cOutFile << std::endl << "[process overall]" << std::endl << std::endl;
00203       ::rusage tResourceUsage;
00204 #ifdef __APPLE__
00205       getrusage(RUSAGE_SELF, &tResourceUsage);
00206 #else
00207       ::getrusage(::RUSAGE_SELF, &tResourceUsage);
00208 #endif
00209       DumpResourceUsageHumanReadable(m_cOutFile, tResourceUsage);
00210       if(! m_vecThreadResourceUsage.empty()) {
00211          for(size_t i = 0; i < m_vecThreadResourceUsage.size(); ++i) {
00212             m_cOutFile << std::endl << "[thread #" << i << " overall]" << std::endl << std::endl;
00213             m_cOutFile << "CPU usage: " << CPUUsage(m_vecThreadResourceUsage[i], fElapsedTime) << "%" << std::endl;
00214             DumpResourceUsageHumanReadable(m_cOutFile, m_vecThreadResourceUsage[i]);
00215          }
00216       }
00217    }
00218 
00219    /****************************************/
00220    /****************************************/
00221 
00222    void CProfiler::FlushAsTable() {
00223       double fStartTime = TV2Sec(m_tWallClockStart);
00224       double fEndTime = TV2Sec(m_tWallClockEnd);
00225       double fElapsedTime = fEndTime - fStartTime;
00226       ::rusage tDiffResourceUsage = m_tResourceUsageEnd - m_tResourceUsageStart;
00227       m_cOutFile << "ProfPortion " << fElapsedTime << " "
00228                  << CPUUsage(tDiffResourceUsage, fElapsedTime) << " ";
00229       DumpResourceUsageAsTableRow(m_cOutFile, tDiffResourceUsage);
00230       m_cOutFile << std::endl << "PortionStart 0 0 ";
00231       DumpResourceUsageAsTableRow(m_cOutFile, m_tResourceUsageStart);
00232       m_cOutFile << std::endl << "PortionEnd 0 0 ";
00233       DumpResourceUsageAsTableRow(m_cOutFile, m_tResourceUsageEnd);
00234       m_cOutFile << std::endl << "Overall 0 0 ";
00235       ::rusage tResourceUsage;
00236 #ifdef __APPLE__
00237       getrusage(RUSAGE_SELF, &tResourceUsage);
00238 #else
00239       ::getrusage(::RUSAGE_SELF, &tResourceUsage);
00240 #endif
00241       DumpResourceUsageAsTableRow(m_cOutFile, tResourceUsage);
00242       if(! m_vecThreadResourceUsage.empty()) {
00243          for(size_t i = 0; i < m_vecThreadResourceUsage.size(); ++i) {
00244             m_cOutFile << std::endl << "thread_" << i << " 0 "
00245                        << CPUUsage(m_vecThreadResourceUsage[i], fElapsedTime) << " ";
00246             DumpResourceUsageAsTableRow(m_cOutFile, m_vecThreadResourceUsage[i]);
00247          }
00248       }
00249       m_cOutFile << std::endl;
00250    }
00251 
00252    /****************************************/
00253    /****************************************/
00254 
00255    void CProfiler::StartWallClock() {
00256       ::gettimeofday(&m_tWallClockStart, NULL);
00257    }
00258 
00259    /****************************************/
00260    /****************************************/
00261 
00262    void CProfiler::StopWallClock() {
00263       ::gettimeofday(&m_tWallClockEnd, NULL);
00264    }
00265 
00266    /****************************************/
00267    /****************************************/
00268 
00269    void CProfiler::StartCPUProfiler() {
00270 #ifdef __APPLE__
00271       getrusage(RUSAGE_SELF, &m_tResourceUsageStart);
00272 #else
00273       ::getrusage(::RUSAGE_SELF, &m_tResourceUsageStart);
00274 #endif
00275    }
00276 
00277    /****************************************/
00278    /****************************************/
00279 
00280    void CProfiler::StopCPUProfiler() {
00281 #ifdef __APPLE__
00282       getrusage(RUSAGE_SELF, &m_tResourceUsageEnd);
00283 #else
00284       ::getrusage(::RUSAGE_SELF, &m_tResourceUsageEnd);
00285 #endif
00286    }
00287 
00288    /****************************************/
00289    /****************************************/
00290 
00291 }
```

---

Generated on 10 Jul 2018 for ARGoS by 
 1.6.1
